# Smartphone-Based Distributed Data Collection Enables Rapid Assessment of Shorebird Habitat Suitability

**DOI:** 10.1371/journal.pone.0164979

**Published:** 2016-11-09

**Authors:** E. Robert Thieler, Sara L. Zeigler, Luke A. Winslow, Megan K. Hines, Jordan S. Read, Jordan I. Walker

**Affiliations:** 1 U.S. Geological Survey, Woods Hole, Massachusetts, United States of America; 2 U.S. Geological Survey, Middleton, Wisconsin, United States of America; Universidade de Vigo, SPAIN

## Abstract

Understanding and managing dynamic coastal landscapes for beach-dependent species requires biological and geological data across the range of relevant environments and habitats. It is difficult to acquire such information; data often have limited focus due to resource constraints, are collected by non-specialists, or lack observational uniformity. We developed an open-source smartphone application called iPlover that addresses these difficulties in collecting biogeomorphic information at piping plover (*Charadrius melodus*) nest sites on coastal beaches. This paper describes iPlover development and evaluates data quality and utility following two years of collection (*n* = 1799 data points over 1500 km of coast between Maine and North Carolina, USA). We found strong agreement between field user and expert assessments and high model skill when data were used for habitat suitability prediction. Methods used here to develop and deploy a distributed data collection system have broad applicability to interdisciplinary environmental monitoring and modeling.

## Introduction

The coastal zone is a highly dynamic environment that changes in response to a variety of short- and long-term processes, such as storms, natural vegetation succession, anthropogenic modifications, climate change, and sea-level rise. However, the range of physical and biological responses of beach environments to climate change and sea level rise is poorly understood at the temporal and spatial scales required for decision making [1]. Similarly, the cumulative impacts of physical and biological change on the quantity and quality of coastal habitats are not well understood, which poses a challenge for natural resource management [2].

Observations of species, their local context, and population dynamics are necessary to understand and predict changes in habitat availability and utilization [3]. Many shorebird species, including the piping plover (*Charadrius melodus*), utilize habitats found on coastal beaches. The piping plover was listed as threatened along the U.S. Atlantic coast in 1986 [4]; recent estimates place the population at fewer than 2000 pairs [5]. As a federally listed species, the conservation and recovery of this species is administered under provisions of the Endangered Species Act of 1973 and includes both population- and habitat-level management recommendations. Because of the dynamic nature of piping plover habitat, the efficacy of future conservation resource allocation and management can be improved by understanding which actions are most likely to increase the persistence and resilience of sensitive coastal habitats. Thus, ongoing species recovery efforts can benefit from information about the future distribution and attributes of plover breeding habitat, particularly under potential threats like sea-level rise and coastal engineering.

**Fig 1:**
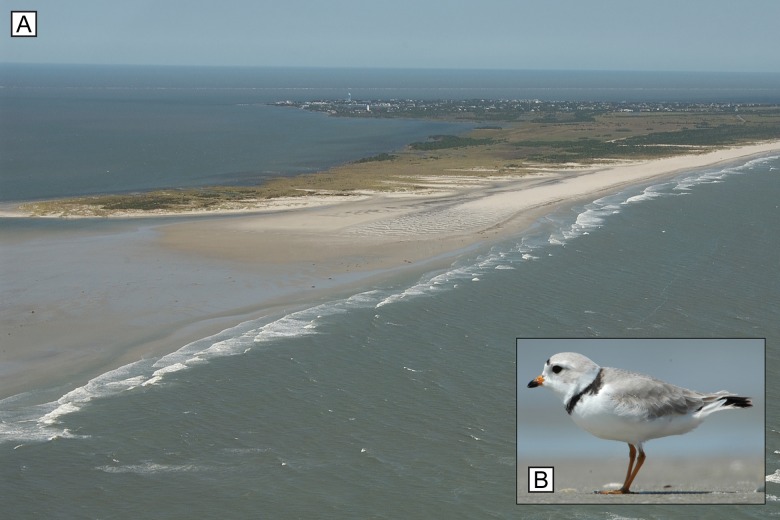
Barrier islands are a principal habitat type in the U.S. Atlantic coast piping plover breeding range. (A) Oblique aerial photograph of southwestern Ocracoke Island, North Carolina, showing open-ocean sandy beach (right), dunes, backbarrier bay, and various types of dune, shrub, forest, and marsh vegetation. (U.S. Geological Survey/photo by Karen L.M. Morgan.) (B) The piping plover (*C*. *melodus*), a federally listed beach-nesting shorebird. (U.S. Fish and Wildlife Service/photo by Gene Nieminen.)

Atlantic coast piping plover nest sites are typically found on low-lying beach and dune systems (Fig 1; [6, 7]). These birds and their habitats respond rapidly to coastal processes like sediment overwash, inlet formation, and island migration [8] that are sensitive to climate-related changes in storminess and the rate of sea-level rise. The piping plover may also serve as a surrogate species for other beach-nesting shorebirds (e.g., American oystercatcher, *Haematopus palliates*; least tern, *Sternula antillarum*; black skimmer, *Rynchops niger*) as well as several federally listed birds (e.g., rufa red knot, *Calidris canutus rufa*), plants (e.g., seabeach amaranth, *Amaranthus pumilus*), and insects (e.g., northeastern beach tiger beetle, *Cicindela dorsalis dorsalis*). Therefore, the ability to predict piping plover habitat availability in the face of climate change can have broad applicability to a variety of coastal species.

As part of the species’ recovery plan [9], piping plovers are observed by a large number of trained monitors throughout their geographic distribution [10]. Monitors represent federal and state agencies, nongovernmental organizations, and land-owning trusts and possess varying levels of expertise in environmental assessment and use of specialized equipment (e.g., global navigation satellite system receiver, GNSS).

Acquiring data needed to quantify and understand piping plover biogeomorphic preferences for nesting habitat required a tool designed to handle diverse users and challenging conditions. Data needed to be standardized and collected synoptically in a harsh environment that includes bright sun, windblown sand, and salt spray. Such data collection often requires multiple prohibitively expensive devices. Thus, a satisfactory solution for data collection along ~1500 km of coastline required a low-cost but adequate device with multiple sensors that could operate in a demanding environment. In addition, an analysis method was necessary that could accommodate a dataset with varying levels of accuracy and subjective observations of habitat characteristics.

Here we describe the development, initial field deployment, and subsequent modification of a smartphone application called iPlover that supports investigations of the effects of coastal change on piping plover habitat availability and utilization. This work is one component of a larger research and management program that seeks to understand and sustain ecological value, ecosystem services, and habitat suitability of beaches in the face of storm impacts, climate change, and sea-level rise.

## Materials and Methods

We describe our materials and methods in two parts. First, we describe the software and other infrastructure used to collect the field data. Second, we describe our approach to evaluating data quality and utility for habitat suitability studies.

### Software and related infrastructure

Data collection requirements for iPlover were defined as: 1) site identification, 2) date and time of observation, 3) geographic location, 4) a photographic image of the site, and 5) a simple biogeomorphic landscape classification to characterize site geomorphology and vegetation. Important operational requirements included the abilities to: 1) perform data collection quickly at a nest site to minimize disturbance and the potential for predator cueing; 2) support offline operation in areas with intermittent or non-existent cellular network coverage; and 3) minimize the time between data collection and application in habitat utilization research models. The specific data inputs and sources are shown in Table 1.

**Table 1:** Data collected in the iPlover application and source of input within the smartphone device.

Data Type	Source
Site identification	Manual (alphanumeric keyboard entry)
Timestamp	Device (clock)
Location and accuracy	Device (GPS)
Site photograph	Device (camera)
Geomorphic setting	Manual (choose from radio button list)
Substrate type	Manual (choose from radio button list)
Vegetation type	Manual (choose from radio button list)
Vegetation density	Manual (choose from radio button list)
Notes	Manual (alphanumeric keyboard entry)

iPlover was built using an agile software development model [11]. This approach ensured: 1) that scientists and developers established and maintained close engagement, 2) a short turnaround from ideation to working application, and 3) robustness to changing operational and functional requirements.

To leverage our existing skillsets and expertise in HTML and JavaScript programming, and to support multiple smartphone platforms with a single codebase, we chose to develop iPlover initially as a web application. Developing web applications (web apps) for mobile devices can be challenging, as capability varies between phones from different manufacturers and different operating system versions. To control project risk in the first season of web app deployment, we limited device support to iOS 8 and its Safari browser (e.g., Apple iPhone 5S). This enabled developers to focus on creating a functional web app in a single testing environment without introducing other devices, operating systems, and browser nuances.

**Fig 2:**
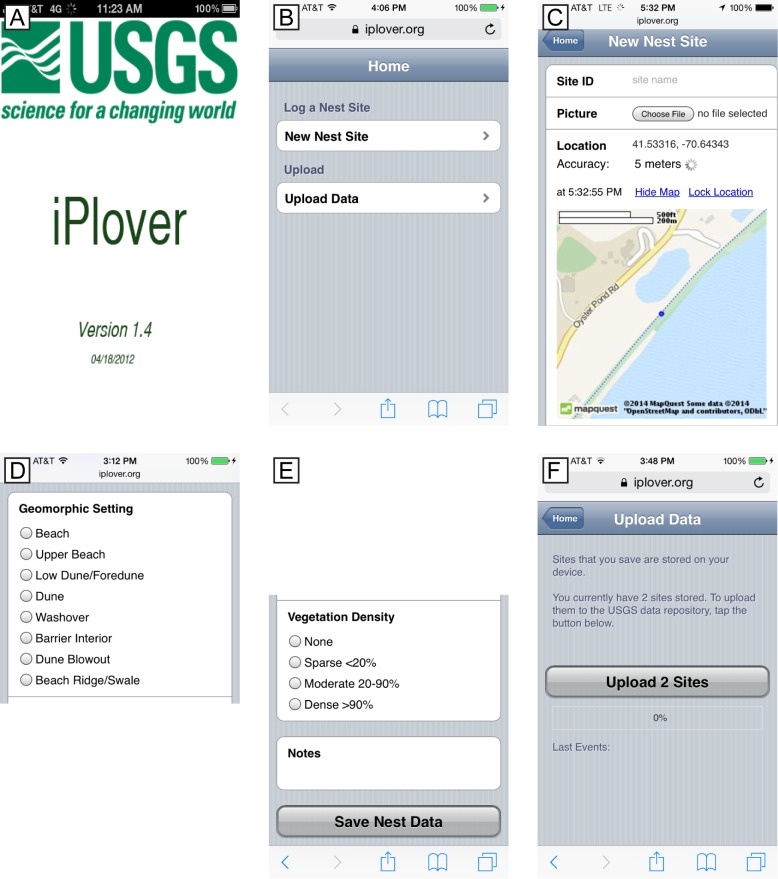
Screenshots showing the iPlover web application interface used during summer 2014. (A) Splash screen showing application name and version. (B) Home page with options to record a new observation ("nest site") or upload existing data. (C) Upper portion of the New Nest Site data entry page, showing site identification entry, photo capture button, and location functionality. (D) Middle portion of the New Nest Site data entry page, showing radio button selection options for the site’s geomorphic setting. (E) Lower portion of the New Nest Site data entry page, showing radio button selection options for vegetation density, the notes field, and "save" button. (F) Upload data page, showing number of sampling data points currently stored on the device, a button to initiate data transfer, and a progress bar to indicate upload status.

We developed a simple interface that either hid unnecessary technical details or presented them in a familiar way (Fig 2). The web app was accessed using the device browser at the app URL. Upon starting the app, a brief splash screen (Fig 2A) indicated the web app version number. The Home page (Fig 2B) presented two basic functions to the user—New Nest Site and Upload Data.

Tapping the New Nest Site button brought up the data input screen (Fig 2C), and the smartphone’s built-in geolocation sensor automatically determined the geolocation of the phone. The geolocation sensor gathered the location information for 60 seconds and reported its accuracy to the user. A field protocol provided to the users specified ~5 m accuracy as acceptable, and users could tap Lock Location to save the current geolocation. If the accuracy was worse than 15–20 meters, the field protocol instructed them to tap a Refresh button and then Lock Location when satisfactory accuracy was achieved. The user could also tap a Show Map link to display a small map with a geolocation accuracy ring to ensure appropriate geolocation.

The other elements of the data entry form (Fig 2D and 2E) were completed by the user and were composed of typical HTML form elements with which web users are generally familiar—text input elements and radio button options. These included fields for nest Site ID, Geomorphic Setting, Substrate Type, Vegetation Type and Vegetation Density. The selections for the biogeomorphic characterization are based on standard classifications (e.g., [12]) and previous work [13, 14].

According to field protocol, a photographic image of the nest site was obtained approximately five meters from the nest to facilitate later evaluation by subject-matter experts. Tapping the Choose File button enabled the smartphone’s camera and standard controls for taking and saving the image into the data form. Finally, a “Notes” field was provided so that the user could provide supplemental information about the site using standard keyboard entry.

To save the data locally to the device, the user tapped the Save Nest Data button. This action also triggered a test for record completeness in required fields (all but Notes). If required data were missing, the user was presented with a validation error containing the names of missing element(s). After a successful save, the app returned to the Home page.

The user submitted locally stored nest site data to a centralized database when a stable internet connection was available. Tapping the Upload Data option from the Home page brought the user to a simple screen (Fig 2F) displaying a summary of sites/records stored on that device as well as a button (Upload *n* Sites) to begin data submission. Beneath the upload button was a progress bar that indicated the status of the uploads, followed by a summary of past events, if any, such as a list of records inserted or errors previously encountered during the upload process.

Piping plover nesting often occurs in areas that lack adequate cellular coverage, requiring offline collection and data storage in the web app. Support for offline functionality required several non-traditional web app implementations. At the most basic level, the in-device browser was instructed to cache all html, style, and JavaScript files as a unit by using a cache manifest file. The manifest specifically described all files necessary for offline operation in the field. Users were instructed to visit and test the website before leaving for the field to ensure the app had been cached.

Storing data, including images, locally within the browser added an additional challenge. The collected nest site data, excluding the image, were stored in a serialized JSON (JavaScript Object Notation; http://www.json.org/) object using the commonly supported localStorage persistent storage functionality (http://dev.w3.org/html5/webstorage/). However, local storage of images was inadequate due to a 10 MB per domain file size limit in Safari for iOS 8. To obviate this limit, the images were stored instead as hex-encoded strings in a Web SQL table (https://www.w3.org/TR/webdatabase/). Web SQL allows 50 MB of storage per domain.

When the user returned to an area with an adequate internet connection, iPlover could be uploaded to the server that hosted the centralized database. Data upload used the HTTPS protocol to sequentially send the data for each site to the server via POST. On the server, images were stored and site data were populated in a PostgreSQL database table. iPlover reported either success or specific errors encountered (e.g., due to poor connection or authentication issues) back to the user following upload.

The web app and centralized database were hosted in the shared U.S. Department of the Interior virtualized server solution. Using the virtual servers, we were able to quickly create instances to accommodate the needs for our web app. This solution was preferred over a more traditional project-based physical server for several reasons. First, we could more rapidly make a virtual server functional. Second, connectivity was simplified on a virtual server compared to a physical server within a more controlled local office IT environment, which could lead to access failures (e.g., firewalls, changes to network configuration). Finally, the virtual server reduced potential downtime due to single-point infrastructure or software failures. The virtual machine instance was set up with the necessary Apache Tomcat and PostgreSQL components, and periodic snapshots were archived as backups. Access was restricted to only a limited set of IPs for administration as part of security protocols.

To control access to iPlover, we used SSL Client Certificate authentication. To access the web app, users were required to have a certificate generated by the server installed in the user profile on their device. This enabled controlled access to the web app by trained and vetted collectors as well as the ability to track the origin of each data record for quality control. We generated self-signed certificates on a production Apache Tomcat server that were distributed via email and installed by users on their devices before entering the field. Passwords necessary for installing the certificate were shared with users while they were trained in the field use of iPlover.

We conducted a 90-minute web-based training session for iPlover users to describe the field protocol we developed to capture and manage data. We also supplied graphical reference documentation with examples of biogeomorphic classification types. A video recording of the training session was made available, and a PDF version of the training materials was provided that could be viewed on the smartphone (e.g., in the iBooks app) or elsewhere for reference in field or office. The users were encouraged to test the app as much as necessary to become comfortable with the interface. A centralized email address was provided for participants to contact scientists and application developers with questions or problems.

Field activities were conducted by the landowners' staff biologists or under pre-existing agreements (e.g., special use permits, memorandums of understanding) with the landowners to conduct piping plover monitoring and management activities. In brief, these biologists used iPlover to characterize the biological and geomorphic characteristics of a 25 m^2^ area around all nests encountered during the course of monitoring as well as around random point coordinates that we disseminated. Because our study leveraged the existing monitoring efforts of our partners, sampling strategies for documenting nest presence were determined by those partners. In the majority of cases, these individuals conducted comprehensive surveys of their sites, meaning that 95–100% of known or recently occupied breeding locations as well as locations that appeared suitable for breeding were surveyed repeatedly in May through July. In instances where sites could not be visited repeatedly, sites were surveyed at least once (at all known or suspected breeding locations) during a nine-day period standardized for the entire Atlantic coast population [10]. The specific number of random points visited each year at a given site was determined by the number of nests observed at that site in the previous year (minimum 5 points). Random point locations were generated randomly within the confines of the site’s boundary using ArcGIS (version 10.3; Esri, http://www.esri.com/). iPlover data collection was restricted to the piping plover breeding season, typically late March through July, so that vegetation and geomorphologic characteristics reflected the period during nest site selection. Under our field protocol, nest site characteristics were recorded using iPlover as soon as practicable after the nest was discovered in the course of monitoring efforts.

### Data evaluation

The science problem underlying the iPlover app was defined by previous work that characterizes and predicts landscape change (e.g., [14, 15]) and piping plover habitat utilization [13]. These approaches used Bayesian networks (BN), which implement fundamental principles of prediction and data assimilation which are well-suited to uncertain observations that can occur in the input data (e.g., geolocation) and subjective classifications (e.g., biogeomorphic characterization) used here. The BN approach provides a framework for applying Bayes’ rule [16, 17] and, in the context applied here, also facilitates application in research models that predict habitat characteristics and utilization.

To evaluate the quality of smartphone geolocation data, we used a SpectraPrecision SP80 GNSS receiver to collect horizontal location data at 44 piping plover nest points in Virginia and Massachusetts. We determined the horizontal difference between the geolocation data obtained from iPlover (i.e., the smartphone’s built-in geolocation functionality; Table 1) with high-resolution nest location data collected with the GNSS receiver. Current smartphones use a hybrid positioning system that combines assisted GPS (A-GPS), wifi, and cellular positioning methods that are generally accurate within 3–8 m [18–20]. GNSS data consist of a combination of real-time kinematic and post-processed positions that are accurate within 3±2 cm in the horizontal. GNSS data are thus two orders of magnitude more accurate than average smartphone geolocation data, so we were able to evaluate smartphone accuracy easily.

We also evaluated the quality and redundancy of subjective biogeomorphic classifications collected in iPlover using a subset of the iPlover dataset collected during the 2014 and 2015 field seasons [21]. We randomly extracted 10% of the iPlover data points (181 records). These points, along with each point’s associated photo and geolocation coordinates, were disseminated to four subject-matter experts, including three coastal geologists and one ecologist. The subject-matter experts were asked to classify the Geomorphic Setting, Substrate Type, Vegetation Type, and Vegetation Density for the 181 records based on each point’s photograph and, for additional context, location in Google Earth (version 7.1.5.1557; Google, https://www.google.com/earth/).

We then compared the classifications for this subset of the iPlover dataset among the four subject-matter experts, and subsequently to those of the iPlover field users, to approximate consistency in iPlover classifications across different users. To compare to the iPlover field classifications, we created a “majority expert” classification for each iPlover test point. In this majority expert classification, the values for Geomorphic Setting, Substrate Type, Vegetation Type, and Vegetation Density for each test point were selected according to the expert consensus. For example, if three of the four experts classified the Geomorphic Setting for a test point as Beach but one expert classified it as Backshore, the majority classification was Beach. We repeated this for every point where at least two of the four experts agreed on the classification. For simplicity in this part of the assessment, we excluded any point where all four experts disagreed on a field’s classification or where no clear consensus could be determined (e.g., two experts classified a Geomorphic Setting as Beach and the other two classified it as Dune). Therefore, fewer than 181 test points were considered when comparing majority expert classifications to the iPlover field dataset (Table 2).

**Table 2:** Classification agreement for the habitat variables considered among four subject-matter experts and between experts and iPlover field users for 181 test points in the iPlover dataset (10% of the full dataset of 1799 data points).

	Geomorphic Setting	Substrate Type	Vegetation Type	Vegetation Density
*Among Experts*				
Total Agreement	60 points	77 points	104 points	91 points
75% Agreement	68	73	48	60
50% Agreement^a^	28	1	15	2
50% Split Agreement^a^	22	30	14	28
No Agreement	3	0	0	0
*Experts compared to iPlover field users*				
Agreement	90 points	121 points	141 points	118 points
No Agreement	66	30	26	35

^a^For 50% agreement, two experts agreed on one classification while the remaining two experts indicated different classifications (for a total of three different classifications). For 50% split agreement, two experts agreed on one classification while the other two experts agreed on a second classification (for a total of two different classifications). Because a majority expert assessment could not be made in the case of 50% split agreement, these points, along with cases where no agreement occurred, were excluded from comparison between the majority expert classification and the original iPlover classification.

Finally, we evaluated the effect that subjectivity in biogeomorphic iPlover classifications had on the skill of a model created to predict piping plover habitat suitability. iPlover was originally designed to collect standardized data that could be used to establish prior probability distributions in a BN for piping plover nesting habitat suitability, similar to one developed recently for Assateague Island National Seashore, Maryland USA [13]. In brief, the BN is composed of four input nodes for Geomorphic Setting, Substrate Type, Vegetation Type, and Vegetation Density and one output node for Habitat Suitability. The output node for Habitat Suitability describes the probability that a location is suitable for piping plover nesting, given that location’s specific combination of habitat characteristics. This model is trained with a dataset that describes these biogeomorphic characteristics at specified locations where a piping plover nest was observed (nest point; assumed high habitat suitability) or where a nest was absent (random point; assumed low habitat suitability). The prior probabilities established through this training dataset then allow a user to make predictions of the likelihood that a given location will be suitable for piping plover nesting given its biogeomorphic characteristics.

To test the effect of subjectivity in the training dataset, we trained the BN with the 181 iPlover test points as classified in field collection and according to the four experts–resulting in five total BNs with different prior probability distributions. We tested each BN’s skill in predicting nest presence (high suitability) or absence (low suitability) in the full iPlover dataset (*n* = 1799 data points). In MATLAB (version R2015b; MathWorks, http://www.mathworks.com/), we generated a 2x2 confusion matrix showing 1) the number of actual nest points that were predicted to be in suitable habitat (i.e., probability habitat was suitale ≥ 0.66; true positives); 2) the number of actual random points that were predicted to be in suitable habitat (false positives); 3) the number of actual nest points that were predicted to be in unsuitable habitat (i.e., probability habitat was suitable ≤ 0.33; false negatives); and 4) the number of actual random points that were predicted to be in unsuitable habitat (true negatives). We considered cases where the BN predicted that the probability of habitat suitability was between 0.33 and 0.66 as “as likely as not” suitable in accordance with the widely used Intergovernmental Panel on Climate Change’s (IPCC) outcome likelihoods [22]. These cases could not be included in error testing, because a suitability prediction could not be attributed to them. However, we considered a high number of “as likely as not” cases indicative of a decline in BN skill and report these numbers accordingly.

Using the confusion matrix, we calculated Cohen’s *kappa* (*K*; [23]) as a measure of the network’s predictive skill, ranging from 0 (poor model performance) to 1 (perfect model performance). We evaluated model performance using the following increments [24]: poor to fair *K* ≤ 0.4; moderate 0.4 < *K* ≤ 0.6; substantial 0.6 < *K* ≤ 0.8; and almost perfect *K* > 0.8.

## Results and Discussion

Here we describe results from the 2014 and 2015 field deployments of the app. We focus on three aspects of this work: 1) initial operational deployment and subsequent app development based on that experience, which refined data collection and improved app usability; 2) evaluation of the acceptability of smartphone geolocation data for our purposes; and 3) evaluation of biogeomorphic characterization data quality and the influence of that quality on habitat suitability predictions.

These data are inherently geospatial and thus evaluating the location and landscape characterization aspects of the data is important. Likewise, it is important to have the ability to conduct such retrospective analyses, rather than accepting all data at face value. The approach we have taken to these evaluations exploits the ability to both mine the data and compare with observations internal (e.g., site photos) and external (e.g., GNSS surveys, aerial imagery) to the app itself.

### Initial Field Deployment

Our initial results are strongly positive across key factors for operational success, including usability, quantitative and descriptive data for nest site characterization, and assimilation into a modeling framework. We were able to quickly obtain a synoptic, low-cost, integrated dataset spanning 1500 km of the U.S. Atlantic piping plover breeding range.

**Fig 3:**
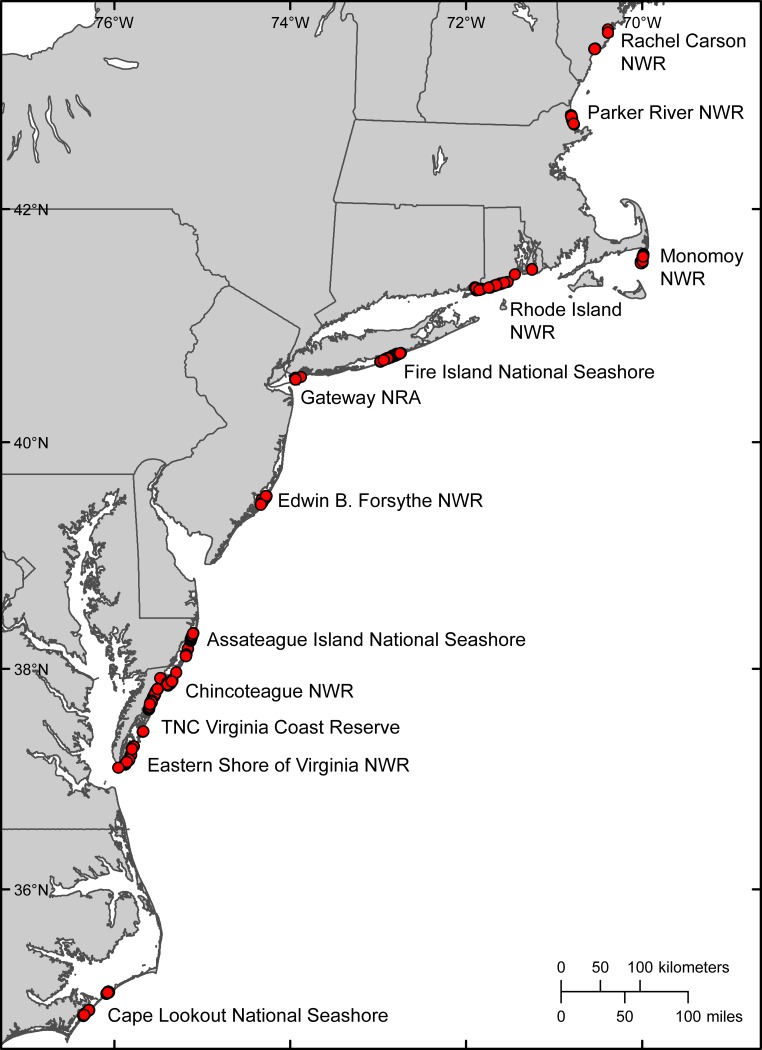
Map showing the locations of iPlover mobile application data collection during summer 2014. Red dots indicate recorded observations (*n* = 574). (Basemap from GSHHG, https://www.ngdc.noaa.gov/mgg/shorelines/gshhs.html.)

In 2014, the iPlover web app was deployed to 12 sites, containing 34 beaches or barrier islands, in the U.S. Atlantic coast piping plover breeding range (Fig 3). Eleven of these sites were on federally managed wildlife refuges, national seashores and recreation areas, and one site was within a private conservation area. These sites are focal points for species and habitat management and include important coastal settings for habitat utilization research. In 2014, field users collected data at 574 locations, which included 492 nest sites. The total also includes a pilot study of 82 random points, which characterized locations where piping plovers did not nest and provided a means to assess how habitat utilization models differentiate suitable and unsuitable habitat [25].

Over the approximately four-month field deployment, field users collected data with the web app. During this time, we received seven requests for assistance with the web app. Five of these requests were related to the app failing to allow data collection when the browser cache limit was reached, and the remaining two requests were related to authentication certificate installation.

**Fig 4:**
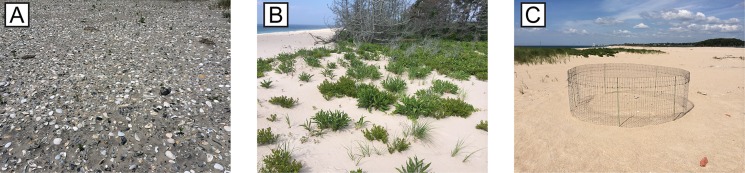
Nest site photos collected using the iPlover web app, showing representative environments. (A) Shell-dominated washover deposit. (B) Moderately vegetated low dune. (C) Open beach with plover nest protected by a wire mesh predator exclosure. Nest site photos were taken by project participants and used for ground-truthing by subject-matter experts.

The form-based interface provided simple, effective usability and appears to have minimized data-entry blunders. For example, we did not receive follow-up requests to modify mis-entered data. In addition, subsequent follow-up data reviews did not identify any mis-entered data. Subjective evaluation of the biogeomorphic classifications by subject-matter experts viewing the high-resolution nest site photos (Fig 4) further aided in the identification of potential blunders or significant mis-classifications (e.g., a site characterized as having no vegetation was shown in the site photograph to have dense vegetation).

During initial field deployment, we found that in-browser storage space limited the number of nest sites that users could save locally on the phone. The images taken by the phone use JPEG compression by default. The coastal images often had complex backgrounds that compressed poorly, resulting in image sizes ranging from 5 to over 11 MB. Thus, the in-browser 50 MB maximum storage capacity was often reached with only a few sites. Once the in-browser storage limit was reached, the app stopped saving new sites.

The self-signed certificates used to control user access were reliable. On the user side, installing a certificate was straightforward but unfamiliar for many smartphone users. Certificate installation also often required that users enter their own device passcode and certificate password several times. We found that the certificate approach was labor-intensive for the developer team to support even 12–15 users and thus would not scale well in a larger data-collection effort due to the amount of time and effort required to generate, distribute, and manage the self-signed certificates.

### Version 2

**Fig 5:**
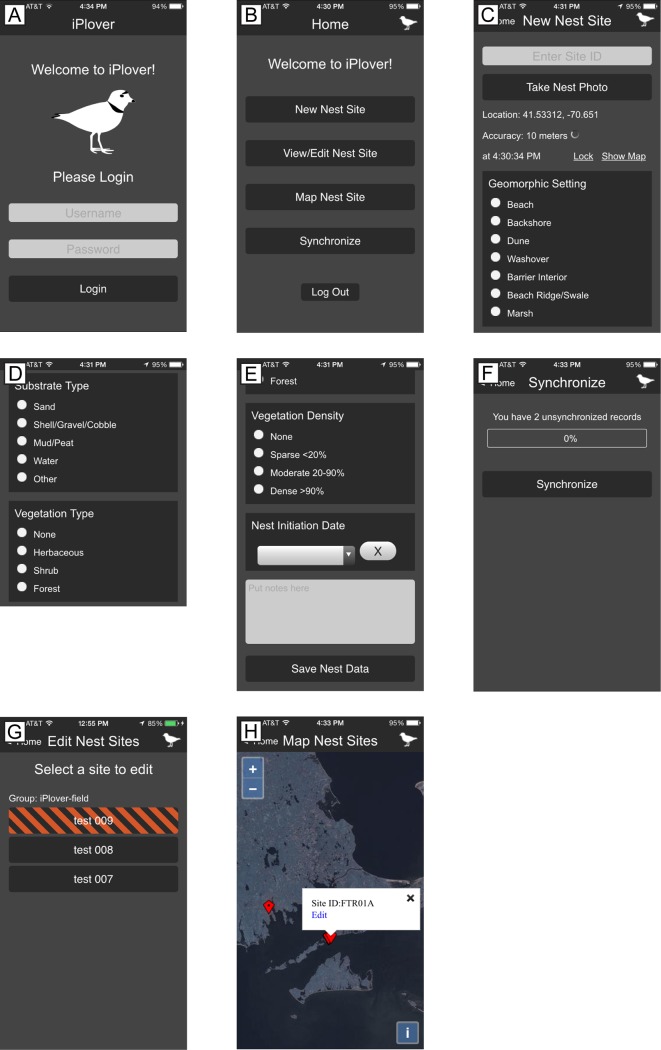
Screenshots showing the iPlover interface developed for native iOS and Android applications. (A) Home page with login screen. (B) Main tasks screen. (C) Upper portion of the New Nest Site data entry page, showing site identification entry, photograph button, location information, and radio button selection options for the site geomorphic setting. (D) Middle portion of the New Nest Site data entry page, showing radio button selection options for substrate and vegetation type. (E) Lower portion of the New Nest Site data entry page, showing radio button selection options for vegetation density, calendar picker for estimated nest initiation date, notes field, and Save Nest Data button. (F) Synchronize data screen, showing number of sites currently stored on the device, button to initiate data transfer, and progress bar to indicate upload status. (G) Edit Nest Sites screen, showing data records available for editing. Records with orange striping indicate data that have not yet been synchronized with the central database. (H) Map Nest Sites screen, showing data records on a map. Tapping a data point produces a pop-up window with the site identifier and an option to edit the data record.

The 2014 field data collection indicated that several changes to the interface would result in more robust and consistent data as well as increased usability (Fig 5). In planning for potentially wider distribution and user participation, we also implemented an email address and password authentication system using identity management infrastructure supplied by the U.S. Geological Survey through my.usgs.gov rather than self-signed certificates. This elegantly supported both Department of the Interior employees using employee credentials as well as iPlover users outside the Department of the Interior (e.g., other federal and state agencies, non-governmental organizations).

In follow-up reviews, the 2014 users requested the ability to review and edit submitted data. This was particularly important for study sites that had multiple smartphones and users collecting data in order to ensure not only complete data collection at a study site but also that the same nest site was not reported multiple times. Therefore, in version 2, we developed a means for field users to view, evaluate, and edit previously collected data points, both in list and map format (Fig 5G–5H). We also implemented a "collection group" system based on study site geography, so that field users would have access only to data collected in their region rather than the entire dataset. This reduced the clutter of the list and map interface as well as the amount of data transfer required between the central database and field user smartphones. The users could retrieve and edit any record collected by a member of their collection group. Because of the distributed nature of the records stored locally on the phones, it was possible to have two different users edit the same record. To deal with this contingency, we used an eventual consistency model [26] where the last write event to the database “wins”.

In version 2 of the application, data continued to be stored locally on the device, but we implemented a "synchronize" function to both upload local data to the central data repository and download nest site data (but not images, to reduce data transfer requirements) to the device. This facilitated the exchange of information among field users at a site and reduced the potential for missing or redundant data.

As described above, a significant limitation in the 2014 field season was robustness due to browser storage limits encountered with high-resolution images. To address this limitation, we chose to move from a web-based application to an installed native phone app, which gives direct and unlimited access to local phone storage. To make this transition quickly (in the ~8 months between field seasons), we used the Apache Cordova platform (https://cordova.apache.org/), a native wrapper for HTML/JavaScript apps, as the basis for building hybrid "native" apps for iOS and Android devices. We chose this approach due to the simplicity of moving the existing codebase into the Apache Cordova framework rather than rebuilding the app using the platform-specific language and Application Programming Interfaces (API). This approach proved to be technologically straightforward and cost-effective in solving the data storage problem. This change, however, introduced additional complexity to the research project, by requiring deployment of the app through third-party app repositories (e.g., Apple App Store, Google Play, Federal Mobile Apps Directory). For the Apple App Store, this required an approximately seven-day turnaround for initial review. Subsequent app updates were generally available in 3–7 days.

We were unable to deploy the Android version to the Google Play distribution platform due to the lack of a current Terms of Service agreement between the U.S. Department of the Interior and Google Inc. However, unlike iOS devices that can only install apps from the Apple App Store, Android devices can be configured to install application packages from other sources. We deployed the Android version of the app to the Federal Mobile Apps Directory (apps.usa.gov) and provided written instructions for Android smartphone users to configure their phones to install the app. This also added complexity to the project, but we did not encounter user technical issues. Overall, the app store (or third-party site) approach was more consistent with typical smartphone apps and thus provided a familiar user experience for accessing, installing, and using the app.

Finally, in response to user feedback from the 2014 season, we reorganized, modified, and added choices for the various biogeomorphic state classifications included in iPlover to better reflect characteristics observed in the field.

In 2015, iPlover field users collected 752 nest and 473 random points using the native iOS or Android version of the app at 19 sites between Maine and North Carolina. This increased the study extent from 34 beaches or barrier islands in 12 sites in 2014 to 83 beaches/barrier islands in 19 sites in 2015. The number of principal participants (those who either or both collected iPlover data or supervised one or more field technicians who collected the data) grew from approximately 16 in 2014 to approximately 35 in 2015.

### Geolocation data

The geolocation capability in the smartphones provided adequate positional accuracy in a short period of time (typically within a few seconds to less than a minute), thus meeting the operational requirement to minimize nest site disturbance.

**Fig 6:**
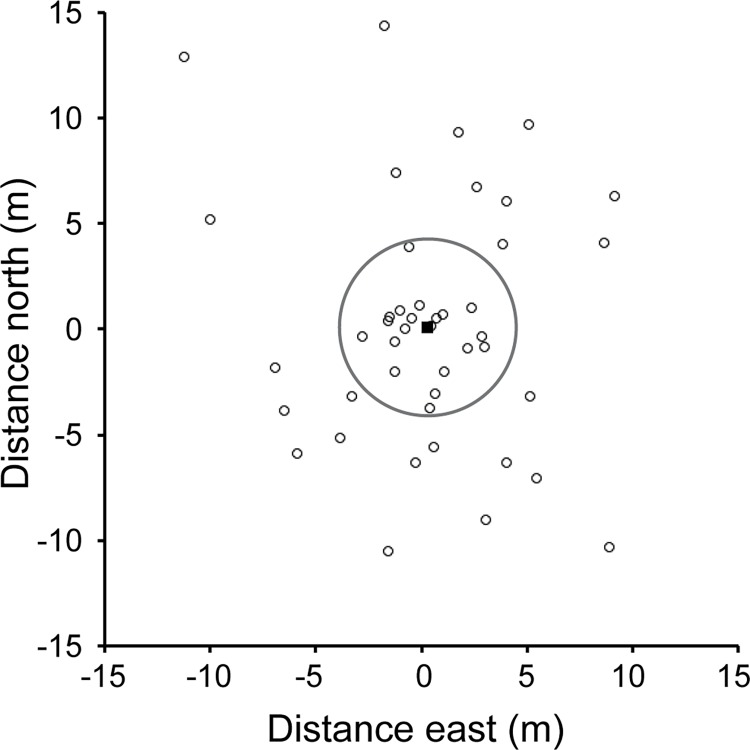
Plot showing spatial difference between global navigation satellite system receiver (GNSS)- and smartphone-derived data points on coastal beaches. Black open circles show the distance between each GNSS observation and the corresponding iPlover observation (S1 Table). The black square is the average distance between the GNSS and the smartphone for the 44 data points, and the gray circle is the one-sigma range around that average distance.

Fig 6 shows the spatial difference between GNSS- and smartphone-derived data for 44 data points collected with iPlover and the corresponding GNSS survey points (data provided in S1 Table). The differences in x (0.3 +/- 4.4 m) and y (0.08 +/- 5.7 m) UTM coordinates are nearly distributed around zero. The average difference between the GNSS points and the smartphones was 5.8 +/- 4.2 m. We did not find spatial variability in accuracy from site to site. For one site where we had repeated measurements at different times on the same day, the difference in geolocation was well within the error reported here.

Although error in iPlover geolocation was relatively small, there were several potential sources of error. First, user movement as he/she acquired location data could have increased error. Second, physical or logistical obstacles (e.g., nest exclosure, Fig 4C; intentional avoidance of nest to prevent predator cueing) could have prevented some users from occupying the nest site precisely. Third, variations in smartphone geolocation accuracy could have occurred due to cellular network or wifi availability. In some heavily developed locations along the U.S. Atlantic coast, dense cellular network and wifi coverage may yield more rapid and accurate geolocation data due to the means by which smartphones determine geolocation [18]. Given that nest sites are located principally on open-ocean beaches, the asymmetric geometry (e.g., cellular towers are alongshore and landward of nest sites, not seaward) of cellular and wifi locations may also result in some compromise of positional accuracy. Finally, error could have arisen from typical GNSS considerations, such as accuracy degradation due to multipath reflections or satellite constellation coverage [19].

Given prior knowledge of the potential issues discussed here, our protocol did not require exact occupation of nest sites. These considerations notwithstanding, the overall accuracy of the geolocation data was sufficient as input into our multi-variate models (e.g., [13, 14]), which are generally robust to uncertain data.

### Biogeomorphic characterizations

We found relatively high redundancy in biogeomorphic classifications by the four experts (S2 Table). At least three of the four experts agreed on their classifications for 71%, 83%, 84%, and 83% of the test iPlover points (*n* = 181) for Geomorphic Setting, Substrate Type, Vegetation Type, and Vegetation Density, respectively (Table 2). However, although there was a clear majority consensus for most of the test points, there remained some level of subjectivity in classifications even among experts.

A majority expert consensus could be determined for 156 of the test iPlover points for Geomorphic Setting; the iPlover field user classification agreed with the majority expert classification for 58% of these points (Table 2; S2 Table). Experts and field users most frequently classified the same point as Backshore, Dune, and Washover, accounting for 91% of the differences among the 5 classifications (Table 3). However, there were no obvious systematic misclassifications for this habitat variable (e.g., a test point was not commonly classified as Backshore by experts and Dune by field users, etc; Table 3). We hypothesize that some of the discrepancy between expert and iPlover user classifications for this variable was due to a lack of context in the iPlover photographs used by experts. iPlover users in the field could evaluate a point’s location relative to neighboring areas and thus had more information when selecting a geomorphic setting. The experts were limited to the information contained in a 25 m^2^ area shown in the iPlover site photograph, and supplemental Google Earth imagery often represented time periods substantially before or after the iPlover data point was actually collected in the field.

**Table 3:** Common differences^a^ in how habitat conditions (Geomorphic Setting, Substrate Type, Vegetation Type, and Vegetation Density) were described according to iPlover field users and subject-matter experts.

iPlover Field User Classification	Majority Expert Classification	Number of Test Points
*Geomorphic Setting*		
Barrier Interior	Dune	7 Points
Ridge/Swale	Dune	2
Backshore	Dune	1
Washover	Dune	3
Barrier Interior	Backshore	2
Beach	Backshore	12
Dune	Backshore	5
Ridge/Swale	Backshore	1
Washover	Backshore	1
Barrier Interior	Washover	13
Beach	Washover	3
Dune	Washover	2
Ridge/Swale	Washover	2
Backshore	Washover	6
*Substrate Type*		
Shell/Gravel/Cobble	Sand	15 points
Sand	Shell/Gravel/Cobble	9
*Vegetation Type*		
None	Herbaceous	12 points
Herbaceous	None	9
*Vegetation Density*		
Sparse	None	13 points
None	Sparse	8
Moderate	Sparse	5

^a^The most common differences between the majority expert classification and the iPlover field user dataset. Differences shown here account for 90%, 86%, 81%, and 74% of the differences for Geomorphic Setting, Substrate Type, Vegetation Type, and Vegetation Density, respectively, between the majority expert classification and the iPlover field user dataset.

For Substrate Type, Vegetation Type, and Vegetation Density, the experts agreed with the iPlover field user classification for 80%, 84%, and 77%, respectively, of the test points for which an expert consensus could be made (Table 2; S2 Table). Systematic errors were more common for these variables compared to Geomorphic Setting (Table 3). What experts classified as Sand was frequently classified as Shell/Gravel/Cobble by iPlover field users and vice versa, accounting for 86% of the differences in this variable. Vegetation Type was frequently classified as Herbaceous by experts and as None by iPlover users and vice versa, accounting for 81% of the differences for this habitat variable. Finally, the most frequent differences in Vegetation Density were between None and Sparse (51% of differences) and between Sparse and Moderate (23%; Table 3).

In summary, the most common differences between the classifications of iPlover field users and experts occurred at the "boundaries" of categorical habitat characteristics. For example, what some experts classified as Backshore, other experts and the field user classified as Beach. If an obvious wrackline or geomorphic feature such as a berm was not visible to separate these two geomorphic settings, points that fell between the ocean and the dune toe could be reasonably characterized as either Beach or Backshore. Experts and iPlover field users also disagreed frequently over whether a point fell in no vegetation or sparse vegetation or whether a point fell within sparse or moderate vegetation cover. Experts and iPlover field users were instructed to visually estimate vegetation density and did not use field-based techniques such as quadrat sampling to quantify density. Therefore, differences in classifications between experts and iPlover field users would be expected in places where vegetation density was close to 20%, separating Sparse from Moderate vegetation, or close to 90%, separating Moderate from Dense vegetation.

Although we observed subjectivity in iPlover biogeomorphic classifications, this subjectivity did not have a major effect on habitat suitability predictions. BN skill remained at *K* = 0.6 irrespective of the expert or iPlover field classification used to parameterize the BN (with one exception; Table 4). The percentage of points where a habitat suitability prediction could not be made fell within 11–16%, depending on which classification was used for training (Table 4). Therefore, we conclude that the probabilistic nature of the BN allowed us to make robust predictions of habitat suitability despite some inconsistency or subjectivity in the dataset used to parameterize the model. In general, our results suggest that BNs offer strong tools for analyzing similar datasets collected by large groups of people, including crowd-sourced datasets, where some subjectivity and observational error in classifications is expected.

**Table 4:** Skill of Bayesian networks used to predict piping plover habitat suitability when trained with data points (*n* = 181) classified separately by four subject-matter experts and by an iPlover user in the field. Models were used to predict nest presence/absence based on the habitat characteristics of 1799 iPlover points collected in 2014 and 2015. Skill was assessed through Cohen’s *kappa* (0 = poor performance, 1 = perfect performance).

	Cohen's *kappa*^*a*^	Number of Test Points (*n*_*total*_ = 1799) Classified As:				
		True +^b^	True -^c^	False +^d^	False -^e^	Unable to Make Prediction^f^
Expert 1	0.6	1065	245	196	49	244 (14%)
Expert 2	0.6	1193	149	255	6	196 (11%)
Expert 3	0.6	1074	234	207	5	279 (16%)
Expert 4	0.6	1100	192	209	4	294 (16%)
iPlover Field User	0.6	1065	237	196	15	286 (16%)

^a^A *kappa* value of 0.4–0.6 indicates moderate model performance.

^b^Number of points that were predicted to be nests sites that were actually observed as nest sites.

^c^Number of points that were predicted to be random points that were actually observed as random points.

^d^Number of points that were predicted to be nest points that were actually observed as random points.

^e^Number of points that were predicted to be random points that were actually observed as nest points.

^f^Number of points where the model predicted that a point was “as likely as not” to be a nest point (or suitable for nesting). Higher numbers represent lower model skill.

### Overall experience

As noted in recent work [27, 28], smartphones and their supporting telecommunications infrastructure have greatly improved and simplified distributed data collection. Functions including geolocation, camera, cacheable forms, data storage, and data transmission are readily harnessed to facilitate data gathering and management. In addition, smartphone interfaces and functionality are used increasingly by people in daily life (e.g., interactive maps, web browsing, radio button selections, built-in camera). In this study, the general lack of technical support requirements and high volume of data collected indicated high usability; users had good facility with smartphone technology and could easily use a simple app. Our work also demonstrates that this low-cost sensor can be configured to collect robust scientific data in a challenging field-setting by non-specialists. This is consistent with smartphone applications used in a variety of field, classroom, and clinical settings (e.g., [29, 30]).

Moving from a web app to an installed Cordova hybrid app improved functionality. Access to the native API provided nearly unlimited storage space, constrained only by the storage space available on the phones. Native and hybrid apps can also offer interactive features that are currently difficult if not impossible to create in a web app. With our Cordova hybrid app, there was also a clean division between client and server. In this case, the server no longer needed to serve HTML content and was simply an access-controlled web service API for submission and retrieval of the observed plover nesting sites and random points. This, along with the virtual machine hosts, kept overhead for the hosting and support of the server-side infrastructure low. Once submitted and accepted in the app store, Apple supported distribution of the app itself, and we used an existing federal government platform to distribute the Android version.

The open-source implementation of iPlover allowed us to control development and licensing costs. This implementation also provided a foundation on which other projects can re-purpose the code base. Examples include data collection for other species in a different ecological environment, or any other application that requires geolocation, imagery, descriptive classification data, and an infrastructure that can be hosted in the cloud. The code is readily adaptable to other field or laboratory data collection needs with reasonable effort to customize data entry forms. The code used for the web and Cordova hybrid app versions are freely and publicly available via GitHub at https://github.com/usgs/iplover. This approach to source code access and distribution increases the level of openness and reproducibility of scientific software [31].

The certificate-based authentication scheme used in the initial field deployment was a quick and lightweight solution. It was adequate for the small number of users involved in the 2014 field season. After the 2014 season, changes to the authentication scheme were made to accommodate a larger number of users with an email-address-based approach that was more familiar to users and better scaled to larger participant numbers.

iPlover provided a common tool and protocols that standardized data collection and quality control and thus facilitated scientific research. The app greatly streamlined data collection, aggregation, and management across widely dispersed sites. The risk of data entry error was low due to the controlled selection options presented to the user (e.g., Figs 2 and 5), and opportunities for transcription errors as data were copied from field journals to computer databases were removed. Furthermore, data collected through iPlover were available for use almost instantaneously, removing common processing time-lags data are collected on paper or through other non-integrated devices.

The overall approach to iPlover data collection, management and use follow principles of the scientific data lifecycle (e.g., [32]). For example, the app reduced time required to acquire and process data, including: 1) data validation, description, and preservation through automated collection of geolocation coordinates, 2) evaluations of data completeness, and 3) data transmission to a stable central repository.

The approach to application development and distributed data collection we employed here can increase positive engagement and interactions between research scientists, field scientists and technicians, and coastal managers [33]. In particular, the collaborative “ownership” and understanding of the overarching science problem—predicting the probability of future habitat suitability under different coastal change scenarios—informed the development of scientific goals and data collection tools. The output of the data collection was thus targeted for providing decision guidance from the outset. This is similar to approaches used in structured decision-making and adaptive management [34, 35]. iPlover is thus a specific example of a generalizable approach to distributed monitoring of physical and ecological systems that supports research, management, education, and outreach that can enable better stewardship of the coastal environment.

## Conclusions

Smartphones enable the rapid collection of data for environmental prediction at large spatial scales. We used an incremental approach to develop an open-source web app and subsequent Cordova hybrid "native" app that allowed us to quickly and efficiently gather a large dataset that is used as input to models that predict habitat suitability for a federally listed beach-nesting species, the piping plover. Evaluations of the data for geospatial and habitat characterization accuracy showed that this form of data collection is robust and skillful. Our experience with iPlover has also provided new insights into the technological, administrative, and scientific benefits of smartphone-based data collection to improve scientific understanding and prediction of habitat utilization at large spatial scales that has broad relevance to other applications, particularly those that use distributed data collection techniques.

## Supporting Information

S1 Table Differences in geolocation data collected at 44 sites using the smartphone geolocation sensor and a high-resolution Global Navigation Satellite System (GNSS) receiver. (DOCX)

S2 Table Independent subject-matter expert and iPlover field user classifications for 181 iPlover test points (10% of total 1799 data points in 2014–2015 dataset). (ODS)
