# Supplementary material for: Smartphone-Based Distributed Data Collection Enables Rapid Assessment of Shorebird Habitat Suitability
Source: PLoS One. 2016 Nov 9;11(11):e0164979. doi: 10.1371/journal.pone.0164979 (PMC5102412; doi:10.1371/journal.pone.0164979)
Supplement: S1 Table — (DOCX) [file pone.0164979.s001.docx]

Smartphone-based distributed data collection enables rapid assessment of shorebird habitat suitability

E. Robert Thieler^1^*, Sara L. Zeigler^1^, Luke A. Winslow^2^, Megan K. Hines^2^, Jordan S. Read^2^, and Jordan I. Walker^2^

^1^ U.S. Geological Survey, Woods Hole, Massachusetts, United States of America

^2^ U.S. Geological Survey, Middleton, Wisconsin, United States of America

* Corresponding author

E-mail: rthieler@usgs.gov (ERT)

**Supplementary Table S1.** Location of data points used to evaluate accuracy of smartphone geolocation data on coastal beaches.

| Date^1^ | Location | Site ID | iPlover X (UTM m) | iPlover Y (UTM m) | iPlover Accuracy^2^ (m) | GNSS X (UTM m) | GNSS Y (UTM m) | GNSS RMS | Δ X (m) | Δ Y (m) | Δ total (m) |
| --- | --- | --- | --- | --- | --- | --- | --- | --- | --- | --- | --- |
| 06/18/2015 | Cedar Island, VA | SCI0214 | 445951.43 | 4163990.19 | 5 | 445949.84 | 4163990.60 | 0.027 | -1.59 | 0.41 | 1.64 |
| 06/18/2015 | Cedar Island, VA | SCI0315 | 446001.39 | 4164083.98 | 5 | 446003.58 | 4164083.07 | 0.064 | 2.19 | -0.91 | 2.37 |
| 06/18/2015 | Cedar Island, VA | SCI1015 | 445767.74 | 4163180.20 | 5 | 445763.86 | 4163175.02 | 0.051 | -3.88 | -5.18 | 6.47 |
| 06/18/2015 | Cedar Island, VA | SCI1415 | 446179.32 | 4164248.00 | 5 | 446184.38 | 4164257.68 | 0.044 | 5.06 | 9.68 | 10.93 |
| 06/18/2015 | Cedar Island, VA | AMOY-SCI1415 | 446186.38 | 4164264.84 | 5 | 446195.03 | 4164268.93 | 0.063 | 8.65 | 4.08 | 9.57 |
| 11/22/2015 | Falmouth, MA | c-gr | 362619.40 | 4606449.65 | 5 | 362617.62 | 4606464.04 | 0.009 | -1.77 | 14.39 | 14.50 |
| 11/29/2015 | Falmouth, MA | c-gr | 362615.88 | 4606454.72 | 5 | 362617.62 | 4606464.04 | 0.009 | 1.75 | 9.32 | 9.48 |
| 11/22/2015 | Falmouth, MA | c-p1 | 362608.34 | 4606502.06 | 5 | 362602.44 | 4606496.18 | 0.009 | -5.90 | -5.88 | 8.32 |
| 11/29/2015 | Falmouth, MA | c-p1 | 362602.57 | 4606495.09 | 5 | 362602.44 | 4606496.18 | 0.009 | -0.13 | 1.10 | 1.11 |
| 12/06/2015 | Falmouth, MA | c-p1 | 362602.74 | 4606502.52 | 5 | 362602.44 | 4606496.18 | 0.009 | -0.30 | -6.33 | 6.34 |
| 11/22/2015 | Falmouth, MA | c-p2 | 362632.88 | 4606474.60 | 5 | 362635.92 | 4606465.59 | 0.010 | 3.04 | -9.01 | 9.51 |
| 11/29/2015 | Falmouth, MA | c-p2 | 362633.05 | 4606465.96 | 5 | 362635.92 | 4606465.59 | 0.010 | 2.87 | -0.36 | 2.90 |
| 11/22/2015 | Falmouth, MA | c-p3 | 362675.36 | 4606311.80 | 5 | 362679.37 | 4606317.84 | 0.008 | 4.01 | 6.04 | 7.25 |
| 12/06/2015 | Falmouth, MA | c-p3 | 362678.70 | 4606317.30 | 10 | 362679.37 | 4606317.84 | 0.008 | 0.67 | 0.53 | 0.86 |
| 12/13/2015 | Falmouth, MA | c-p3 | 362690.62 | 4606304.97 | 5 | 362679.37 | 4606317.84 | 0.008 | -11.25 | 12.87 | 17.09 |
| 11/22/2015 | Falmouth, MA | c-p4 | 362747.62 | 4606154.10 | 10 | 362737.64 | 4606159.30 | 0.010 | -9.99 | 5.20 | 11.26 |
| 12/06/2015 | Falmouth, MA | c-p4 | 362738.69 | 4606158.41 | 5 | 362737.64 | 4606159.30 | 0.010 | -1.05 | 0.89 | 1.38 |
| 12/13/2015 | Falmouth, MA | c-p4 | 362744.56 | 4606161.14 | 5 | 362737.64 | 4606159.30 | 0.010 | -6.93 | -1.84 | 7.17 |
| 11/22/2015 | Falmouth, MA | c-sp | 362639.69 | 4606465.58 | 5 | 362643.56 | 4606469.63 | 0.009 | 3.87 | 4.05 | 5.60 |
| 11/29/2015 | Falmouth, MA | c-sp | 362644.35 | 4606469.62 | 5 | 362643.56 | 4606469.63 | 0.009 | -0.79 | 0.01 | 0.79 |
| 11/29/2015 | Falmouth, MA | c-sp | 362644.81 | 4606471.61 | 10 | 362643.56 | 4606469.63 | 0.009 | -1.25 | -1.98 | 2.35 |
| 07/16/2015 | Martha's Vineyard, MA | MVDFB4A | 348331.04 | 4579939.33 | 5 | 348332.09 | 4579937.32 | 0.024 | 1.04 | -2.00 | 2.26 |
| 07/16/2015 | Martha's Vineyard, MA | MVEGP3A | 369725.84 | 4578855.78 | 10 | 369725.26 | 4578859.71 | 0.016 | -0.58 | 3.93 | 3.97 |
| 07/16/2015 | Martha's Vineyard, MA | MVEGP2B | 369599.05 | 4578871.81 | 5 | 369595.72 | 4578868.61 | 0.013 | -3.33 | -3.20 | 4.62 |
| 07/16/2015 | Martha's Vineyard, MA | MVDFB8A | 347962.19 | 4579722.96 | 5 | 347962.77 | 4579717.35 | 0.056 | 0.58 | -5.60 | 5.63 |
| 07/16/2015 | Martha's Vineyard, MA | MVEGP1A | 370352.79 | 4578854.19 | 5 | 370357.92 | 4578851.00 | 0.014 | 5.13 | -3.19 | 6.04 |
| 07/16/2015 | Martha's Vineyard, MA | MVDFB6A | 348812.20 | 4579910.86 | 5 | 348816.21 | 4579904.55 | 0.014 | 4.01 | -6.31 | 7.48 |
| 07/16/2015 | Martha's Vineyard, MA | MVDFB7A | 348318.52 | 4579946.92 | 10 | 348316.96 | 4579936.43 | 0.051 | -1.56 | -10.49 | 10.61 |
| 07/15/2015 | Monomoy Island, MA | smny36b | 417398.08 | 4601085.19 | 5 | 417398.52 | 4601085.33 | 0.011 | 0.44 | 0.14 | 0.46 |
| 07/15/2015 | Monomoy Island, MA | smny30a | 417358.47 | 4601388.06 | 5 | 417357.22 | 4601387.45 | 0.011 | -1.25 | -0.60 | 1.39 |
| 07/15/2015 | Monomoy Island, MA | smny24a | 416365.59 | 4601655.96 | 5 | 416362.77 | 4601655.63 | 0.014 | -2.82 | -0.33 | 2.84 |
| 07/15/2015 | Monomoy Island, MA | smny17a | 416404.05 | 4601738.82 | 5 | 416407.02 | 4601737.96 | 0.010 | 2.98 | -0.86 | 3.10 |
| 07/15/2015 | Monomoy Island, MA | smny23a | 417354.01 | 4600928.12 | 5 | 417354.65 | 4600925.04 | 0.010 | 0.64 | -3.09 | 3.15 |
| 07/15/2015 | Monomoy Island, MA | smny18a | 416285.17 | 4601635.31 | 5 | 416283.97 | 4601642.71 | 0.015 | -1.20 | 7.40 | 7.49 |
| 07/15/2015 | Monomoy Island, MA | smny23b | 417425.73 | 4601000.97 | 5 | 417419.20 | 4600997.09 | 0.009 | -6.54 | -3.88 | 7.60 |
| 07/13/2015 | Plymouth, MA^3^ | PESLP01a | 372629.46 | 4633103.92 | 4 | 372631.85 | 4633104.94 | 0.067 | 2.38 | 1.01 | 2.59 |
| 07/13/2015 | Sandwich, MA^3^ | SSH06a | 377229.41 | 4624454.73 | 4 | 377228.97 | 4624455.24 | 0.066 | -0.45 | 0.51 | 0.67 |
| 07/13/2015 | Sandwich, MA^3^ | STN03a | 377136.95 | 4624665.07 | 4 | 377135.45 | 4624665.67 | 0.061 | -1.50 | 0.60 | 1.62 |
| 07/13/2015 | Sandwich, MA^3^ | SSH03a | 377938.26 | 4624221.16 | 4 | 377940.85 | 4624227.91 | 0.056 | 2.59 | 6.75 | 7.23 |
| 07/13/2015 | Sandwich, MA^3^ | SSB03c | 373511.04 | 4628181.96 | 4 | 373520.17 | 4628188.28 | 0.076 | 9.13 | 6.32 | 11.11 |
| 07/13/2015 | Sandwich, MA^3^ | STN03b | 377169.28 | 4624696.34 | 4 | 377178.17 | 4624686.01 | 0.041 | 8.89 | -10.33 | 13.63 |
| 06/15/2015 | Wreck Island, VA | Wreck_R2S | 429365.99 | 4122938.57 | 5 | 429367.02 | 4122939.26 | 0.049 | 1.03 | 0.69 | 1.24 |
| 06/15/2015 | Wreck Island, VA | Wreck_R3 | 428815.13 | 4121790.73 | 5 | 428815.54 | 4121786.97 | 0.080 | 0.40 | -3.77 | 3.79 |
| 06/15/2015 | Wreck Island, VA | Wreck_R1S | 429072.31 | 4122132.66 | 5 | 429077.75 | 4122125.57 | 0.037 | 5.44 | -7.08 | 8.93 |

Notes

^1^Date of GNSS survey.

^2^The geolocation API for the iPhone reports accuracy in 5 m increments; Android devices report accuracy with a large number of significant digits, which we round here to the nearest meter.

^3^Android device used to collect iPlover data.
